# Comparison of the Effect of Aerobic Exercise Versus Yoga on Endothelial Function and Insulin Resistance in Type 2 Diabetic Patients

**DOI:** 10.7759/cureus.90578

**Published:** 2025-08-20

**Authors:** Isha Gupta, Ghotekar L.H., Anupam Prakash, Ramesh Aggarwal, Rajiv Bandhu, Rajeev Goyal

**Affiliations:** 1 Internal Medicine, Lady Hardinge Medical College, New Delhi, IND; 2 Physiology, Lady Hardinge Medical College, New Delhi, IND; 3 Biochemistry, Lady Hardinge Medical College, New Delhi, IND

**Keywords:** aerobic exercise, endothelial function, homa-ir, icam-1, insulin resistance, type 2 diabetes mellitus, yoga

## Abstract

Background

Type 2 diabetes mellitus (T2DM) is a common metabolic disorder often linked to endothelial dysfunction and insulin resistance, particularly in individuals with a body mass index (BMI) of 23 kg/m² or higher. This study examines the impact of aerobic exercise and yoga on markers of endothelial function and insulin sensitivity in individuals with T2DM.

Methodology

A quasi-experimental study was conducted involving adults diagnosed with T2DM who met specific inclusion criteria, including age over 18 years, BMI ≥23 kg/m², and HbA1c ≤7.5%. Participants were assigned to one of the following three groups: aerobic exercise, yoga, or a control group. The intervention lasted 12 weeks. Measurements of fasting blood sugar (FBS), HbA1c, insulin resistance (homeostatic model assessment for insulin resistance (HOMA-IR)), and endothelial function by intracellular adhesion molecule-1 (ICAM-1) levels were taken before and after the intervention. Statistical analyses were conducted using t-tests and chi-square tests to assess group differences.

Results

Both the aerobic and yoga groups demonstrated significant reductions in HbA1c, FBS, HOMA-IR, and ICAM-1 levels compared to the control group. Aerobic exercise showed greater improvement in endothelial function, whereas yoga was more effective in enhancing insulin sensitivity.

Conclusions

Regular aerobic or yoga-based exercise interventions are effective non-pharmacological strategies to improve vascular health and insulin resistance in individuals with T2DM. These findings support integrating structured physical activity into diabetes management programs. Tailoring exercise modalities based on patient preference and physical capability may further help reduce diabetes-related complications and improve long-term outcomes.

## Introduction

Diabetes mellitus (DM) is the most prevalent metabolic disease globally, with an estimated prevalence of 589 million adults in 2025, and projections to rise to 853 million by 2050 [1]. Persistent metabolic derangement secondary to hyperglycemia is the defining feature of DM. It primarily comprises type 1 diabetes (insulin deficiency) and type 2 diabetes (insulin resistance or decreased sensitivity) [1]. Epidemiological rates of obesity, a treatable and preventable condition, are strong impelling forces behind the epidemic rise in the incidence of type 2 diabetes mellitus (T2DM), which is largely induced by unhealthy diet, sedentary life, and adipose tissue remodeling [2].

Endothelial dysfunction, a vasoconstrictive, pro-inflammatory, and pro-thrombotic condition caused by impaired endothelial-derived vasodilation, is a common complication of diabetes [3]. Non-pharmacological methods such as smoking cessation, diet, and exercise are recommended lifestyle changes for the treatment of obesity-related diseases, including T2DM [4].

Exercise has the potential as an endothelial protection as well as a repair strategy [5]. Regular interval physical training enhances body composition, cardiovascular function, insulin action, and quality of life [6]. High-intensity training is observed to enhance endothelial function by vasodilation due to increased expression of nitric oxide (NO) and insulin-like growth factor-1 (IGF-1) receptors, as well as atherosclerosis protection even in T2DM patients [7].

Aerobic activities, such as walking, jogging, and cycling, improve insulin sensitivity, vascular health, and aerobic fitness and reduce body fat [8]. The American College of Sports Medicine (ACSM) and American Diabetes Association (ADA) recommend a minimum of 150 minutes/week of moderate-to-vigorous aerobic physical activity, with exercise-induced insulin sensitivity increases remaining up to 48 hours [9,10]. Skeletal muscle plays a pivotal role in the regulation of blood glucose via insulin-independent and insulin-dependent mechanisms, namely, GLUT-4-mediated glucose uptake during exercise [11]. A single bout of exercise has a beneficial effect on insulin sensitivity before weight loss or adaptation [12].

Yoga is an inexpensive, convenient technique that improves glycemic control in non-insulin-dependent DM patients through enhanced pancreatic beta-cell sensitivity and reduced oral hypoglycemic agent intake [13]. Although mechanisms are unclear, neurohormonal modulation with insulin and glucagon is implicated. Homeostasis model assessment of insulin resistance (HOMA-IR) provides a convenient measure to estimate insulin resistance from fasting insulin and glucose, which is necessary for the diagnosis and management of T2DM [14]. Its accuracy can vary depending on fasting or intersubject metabolic differences.

Oxidative stress has a significant role in cardiovascular disorders and diabetes pathogenesis, where high glucose levels generate reactive oxygen species, leading to endothelial and cellular dysfunction [15]. Cytokine-induced endothelial activation leads to increased vascular permeability, procoagulant state, and increased expression of adhesion molecules such as intracellular adhesion molecule-1 (ICAM-1) and vascular cell adhesion molecule-1 (VCAM-1) [16]. The concentration of soluble ICAM-1 (sICAM-1) is elevated during endothelial activation and can be considered a prospective marker of endothelial dysfunction [16]. This study aimed to compare the effects of structured aerobic exercise and yoga, both considered forms of planned, regular physical activity, on glycemic control, endothelial function, and insulin resistance in individuals with T2DM and body mass index (BMI) ≥23 kg/m², following guidelines set forth by the ACSM and ADA.

The primary objective of this study was to compare the effects of aerobic exercise and yoga on endothelial function, measured by ICAM-1, and insulin resistance, measured by HOMA-IR, in adults with T2DM and BMI ≥23 kg/m². The seconadry obective was to compare the effects on BMI, weight, and fasting blood sugar.

## Materials and methods

This quasi-experimental study was conducted in the Department of Medicine, Lady Hardinge Medical College (LHMC) and SSK Hospital, New Delhi, between May 2023 and November 2024. Adults aged≥18 years attending the outpatient department and diabetes clinic constituted the study population. Participants were enrolled if they met all the following inclusion criteria: age ≥18 years, diagnosis of T2DM, BMI ≥23 kg/m², and HbA1c ≤7.5%. Exclusion criteria were patients with exercise contraindications such as ischemic heart disease, heart failure, uncontrolled hypertension, arrhythmias, febrile illness, deep vein thrombosis, arthritis, recent stroke, or hyperthyroidism; patients who were unable to stand or had amputations; and patients requiring a modification in their ongoing treatment for diabetes.

After obtaining informed consent, clinical evaluation was performed, and baseline investigations were conducted, such as ICAM-1 and HOMA-IR. Participants were divided into the following two groups based on non-randomized allocation: an intervention group (Group 1) that performed guided aerobic or yoga exercises, and a control group (Group 2) that performed no instructor-led exercise program. Group 1 was further divided into Group A and Group B. Group A performed 30 minutes of aerobic exercise on five days per week (150 minutes/week), while Group B performed 15 minutes of yoga three to four days per week. Control group participants were instructed to avoid new structured exercise, and self-reported activity logs were used to monitor adherence and exclude those with regular exercise routines.

Age-specific yoga regimens were formulated under professional supervision initially and later as a home routine with regular monitoring. Attendance at sessions was tracked, home practice was monitored by diaries and video recordings, and compliance was encouraged through telephonic reminders every third day. Non-compliance with exercise routines was considered a dropout. The strict monitoring and encouragement of the participants throughout the study led to zero dropouts in the study. Both groups followed their usual diet, medication, and lifestyle routines throughout the 12-week study duration. Post-intervention measurements involved repeating all baseline examinations.

Venous blood samples were collected and processed under aseptic conditions. Insulin was measured using chemiluminescent immunoassay, and ICAM-1 was assessed using an enzyme-linked immunosorbent assay (ELISA)-based assay. Serum samples were stored at -20°C until analysis. The HOMA-IR score was calculated using the following formula: (fasting insulin (µU/L) × fasting glucose (mmol/L))/22.5, with a cut-off of 2.5 for insulin resistance. Compliance was also increased by asking Group A participants to download the Fit India mobile application, maintain exercise records, and submit regular exercise videos. Information was collected on a pre-tested, pre-designed proforma.

The Diacione Human CD54/ICAM-1 ELISA kit is a solid-phase sandwich ELISA for the in vitro qualitative and quantitative determination of sICAM-1/CD54 in supernatants, buffered solutions, or serum and plasma samples. This assay recognizes both natural and recombinant human CD54/ICAM-1. A capture antibody highly specific for CD54/ICAM-1 was coated to the wells of the microtiter strip plate provided during manufacture. Binding of CD54/ICAM-1 samples and known standards to the capture antibodies and subsequent binding of the Biotinylated anti-CD54/ICAM-1 secondary antibody to the analyte were completed during the same incubation period. Any excess unbound analyte and secondary antibody were removed. The horseradish peroxidase conjugate solution was then added to every well, including the zero wells, and following incubation, the excess conjugate was removed by careful washing. A chromogen substrate was added to the wells, resulting in the progressive development of a blue-colored complex with the conjugate. The color development was stopped by the addition of acid, turning the resultant final product yellow. The intensity of the produced colored complex was directly proportional to the concentration of CD54/ICAM-1 present in the samples and standards. The absorbance of the color complex was then measured, and the generated optical density values for each standard were plotted against the expected concentration, forming a standard curve. This standard curve was then used to accurately determine the concentration of CD54/ICAM-1 in any sample tested. The sensitivity or minimum detectable dose of CD54/ICAM-1 using this Diaclone Human CD54/ICAM-1 ELISA kit was found to be 0.1 ng/mL.

The Access Ultrasensitive Insulin assay is a paramagnetic particle, chemiluminescent immunoassay for the quantitative determination of insulin levels in human serum and plasma (EDTA) using the Access Immunoassay Systems. The Access Ultrasensitive Insulin assay is a simultaneous one-step immunoenzymatic (“sandwich”) assay. A sample was added to a reaction vessel along with mouse monoclonal anti-insulin alkaline phosphatase conjugate and paramagnetic particles coated with mouse monoclonal anti-insulin antibody. The serum or plasma insulin was bound to the antibody on the solid phase, while the conjugate reacted with a different antigenic site on the insulin molecule. After incubation in a reaction vessel, materials bound to the solid phase were held in a magnetic field while unbound materials were washed away. Then, the chemiluminescent substrate Lumi-Phos 530 was added to the vessel, and light generated by the reaction was measured with a luminometer. The light production was directly proportional to the concentration of insulin in the sample. The amount of analyte in the sample was determined from a stored, multi-point calibration curve.

The data were analyzed using SPSS version 29.0.0 (IBM Corp., Armonk, NY, USA). Continuous variables were presented as mean ± standard deviation and compared using paired or unpaired t-tests. Categorical variables were expressed as percentages and compared using chi-square tests. Descriptive statistics were expressed as mean ± SD for normally distributed variables and median (IQR) for skewed distributions. Group comparisons were made using parametric or non-parametric tests as appropriate. Tests for normality (e.g., Shapiro-Wilk test) were used to guide the choice of statistical method. Statistical significance was considered at a p-value <0.05. Primary outcome measures were changes in ICAM-1 and insulin resistance (HOMA-IR) following the 12-week intervention. An intention-to-treat analysis was performed.

## Results

The distribution of participants according to the groups indicated that 25.0% of the participants were assigned to Group A, who performed aerobic exercise, and another 25.0% were assigned to Group B, who performed yoga. The remaining 50.0% of the participants were placed in Group 2, the control group, and included participants with a BMI of 23 kg/m² or higher, a diagnosis of T2DM, and no exercise (Table 1).

**Table 1:** Distribution of the participants in the study groups. CI: confidence interval

Group	Frequency	Percentage	95% CI	χ² statistic	P-value
Group A	20	25.0%	16.3%–36.2%	χ² = 13.33	0.0013
Group B	20	25.0%	16.3%–36.2%		
Group 2	40	50.0%	39.3%–60.7%		

The mean (SD) of BMI (kg/m²) (baseline) of Group A was 27.36 (3.11) kg/m². The mean (SD) of BMI (kg/m²) (Baseline) of Group B was 27.90 (3.60) kg/m². The mean (SD) of BMI (g/m²) (baseline) of Group 2 was 27.42 (3.07) kg/m². The median (IQR) of BMI (kg/m²) (baseline) of Group A was 26.31 (25.15-30.41) kg/m². The median (IQR) of BMI (kg/m²) (baseline) of Group B was 26.98 (25.93-28.57) kg/m². The median (IQR) of BMI (kg/m²) (baseline) of Group 2 was 26.47 (25.04-29.3) kg/m². The BMI (kg/m²) (baseline) of Group A ranged from 23.53 to 34.21 kg/m². The BMI (kg/m²) (baseline) in Group B ranged from 23.28 to 37.8 kg/m². The BMI (kg/m²) (baseline) in Group 2 ranged from 23.15 to 35.16 kg/m² (Table 2).

**Table 2:** Association between groups and BMI (kg/m²) (baseline). BMI: body mass index; IQR: interquartile range

Statistic	Group A	Group B	Group 2	Kruskal-Wallis test
Mean (SD)	27.36 (3.11)	27.90 (3.60)	27.42 (3.07)	χ² = 0.508
Median (IQR)	26.31 (25.15–30.41)	26.98 (25.93–28.57)	26.47 (25.04–29.30)	p = 0.776
Minimum–Maximum	23.53–34.21	23.28–37.80	23.15–35.16	

Groups A and B showed improvements in BMI and weight significantly over 12 weeks, whereas Group 2 showed no significant change. Fasting blood sugar (FBS) decreased significantly in Groups A and B, indicating better glycemic control, while FBS in Group 2 decreased minimally. Similarly, insulin levels and HOMA-IR increased significantly in Groups A and B but remained relatively stable in Group 2, indicating increased sensitivity to insulin with interventions. In total, the Group A and Group B interventions significantly improved weight and glucose metabolism, while Group 2 was altered little or not at all, as attested by the highly significant overall p-values for key metabolic markers (Table 3).

**Table 3:** Anthropometric and glycemic parameters. ANOVA: analysis of variance; BMI: body mass index; FBS: fasting blood sugar; HOMA-IR: homeostatic model assessment for insulin resistance

Parameter	Group	Mean (SD), baseline	Mean (SD), 12 weeks	Between-group comparison*(Kruskal-Wallis χ²),*Baseline, 12 weeks	Within-group change*(Wilcoxon or t-test)*	Overall comparison of change*(ANOVA F-statistic)*
BMI (kg/m²)	Group A	27.36 (3.11)	26.65 (2.98)	χ² = 0.508, 0.655	Z = -3.52, p < 0.001	F = 12.84, p < 0.001
	Group B	27.90 (3.60)	27.29 (3.49)		Z = -3.27, p < 0.001	
	Group 2	27.42 (3.07)	27.32 (3.05)		Z = -1.34, p = 0.182	
Weight (kg)	Group A	67.45 (7.21)	65.70 (6.97)	χ² = 0.167, 1.05	Z = -3.59, p < 0.001	F = 10.62, p < 0.001
	Group B	66.65 (9.30)	65.20 (9.08)		Z = -3.34, p < 0.001	
	Group 2	67.58 (8.50)	67.30 (8.30)		Z = -1.53, p = 0.125	
FBS (mg/dL)	Group A	127.00 (12.15)	112.15 (8.29)	χ² = 7.39, 7.91	Z = -3.88, p < 0.001	F = 15.21, p < 0.001
	Group B	134.20 (14.88)	118.85 (12.71)		Z = -3.64, p < 0.001	
	Group 2	123.65 (14.06)	121.17 (12.33)		Z = -2.15, p = 0.031	
Insulin (µIU/mL)	Group A	8.94 (3.37)	7.79 (3.05)	χ² = 2.03, 3.96	Z = -3.41, p < 0.001	F = 8.87, p < 0.001
	Group B	9.84 (2.83)	9.01 (2.53)		Z = -3.12, p < 0.001	
	Group 2	8.94 (3.17)	9.12 (3.02)		Z = -1.51, p = 0.132	
HOMA-IR	Group A	2.80 (1.08)	2.17 (0.91)	χ² = 3.50, 5.39	Z = -3.44, p < 0.001	F = 11.04, p < 0.001
	Group B	3.30 (1.20)	2.68 (0.92)		Z = -3.16, p < 0.001	
	Group 2	2.78 (1.21)	2.76 (1.06)		Z = -0.22, p = 0.826	

Regarding ICAM-1, a marker of inflammation, baseline levels were higher in Group 2 compared with Groups A and B. At 12 weeks, Groups A and B had marked reductions in ICAM-1 levels, indicating an anti-inflammatory effect, while Group 2 had little change. ICAM-1 improved significantly in Groups A and B but not at all in Group 2, highlighting just how effective the treatments were for Groups A and B (Table 4).

**Table 4:** ICAM-1 levels of the study groups. ANOVA: analysis of variance; ICAM-1: intracellular adhesion molecule-1

Group	Mean (SD), baseline	Mean (SD), 12 weeks	Between-group comparison (Kruskal-Wallis χ²), baseline, 12 weeks	Within-group change (Wilcoxon Z)	Overall change comparison (ANOVA F)
Group A	163.5 (2.89)	136.2 (2.41)	χ² = 9.61, p = 0.008; χ² = 20.45, p < 0.001	Z = -3.89, p < 0.001	F = 19.72, p < 0.001
Group B	161.4 (2.88)	144.3 (3.57)		Z = -3.08, p = 0.002	
Group 2	181.6 (2.92)	181.4 (3.10)		Z = -0.28, p = 0.778	

## Discussion

This study compared the effects of aerobic exercise and yoga on primary markers of health among T2DM patients with a BMI ≥23 kg/m² for 12 weeks. Weight, BMI, FBS, and HOMA-IR all improved significantly in both exercise groups. Our findings were consistent with previous studies, such as Ho et al. [17], but also emphasized differences in the magnitude of change. For example, the weight reduction in the yoga and aerobic groups in our study was more pronounced than that reported by Ho et al., where little changes in weight were observed after similar exercise interventions. Antidiabetic drugs also alter the fat and muscle mass in diabetic patients. Monitoring body composition alongside glycemic control is important for comprehensive patient care [18].

Our study also showed significant improvements in insulin resistance, as measured by HOMA-IR, in yoga and aerobic exercise groups. The HOMA-IR score in the aerobic group decreased from 2.8 to 2.17, and in the yoga group, from 3.30 to 2.68. These enhancements were consistent with studies by Yokoyama et al. [19] and Amaravadi et al. [20], which documented similar enhancements following exercise. However, Group 2 participants, the control group, did not change in all parameters, which reinforced the significant effect of exercise compared to those without any structured physical exercise/yoga regimen.

Aerobic exercise lowered ICAM-1 levels significantly, consistent with the study by Nieman et al. [21], who credited decreases in pro-inflammatory cytokines to moderate-intensity aerobic exercise. Yoga reduced ICAM-1 levels but less so than aerobic exercise, consistent with the study by O’Connor et al. [22], who found yoga to reduce inflammatory markers but less so. Insulin resistance promotes weight gain and visceral fat accumulation, triggering endothelial dysfunction [23].

Aerobic exercise increases blood flow and shear stress on the vascular endothelium, which promotes nitric oxide production and suppresses the expression of adhesion molecules such as ICAM-1. This leads to reduced endothelial inflammation and improved insulin receptor signaling. Meanwhile, yoga’s combination of physical movement and breath control modulates the autonomic nervous system by lowering sympathetic output and cortisol levels, thereby decreasing systemic inflammation. These effects collectively reduce ICAM-1 levels, which correlate with improved endothelial function and enhanced insulin sensitivity. Linking these pathways helps explain how both aerobic and yoga exercises contribute to better inflammatory profiles and metabolic outcomes. These results suggest that aerobic as well as yoga training are both responsible for decreasing inflammation, but with stronger effects by aerobic exercise.

Future perspectives

Future studies should explore the effects of combined yoga and aerobic exercise on diabetics. Aerobic exercise improves endothelial function and lowers ICAM-1 through increased blood flow and NO, while yoga reduces inflammation by activating the parasympathetic nervous system and lowering stress hormones. Combined, these effects may synergistically enhance insulin sensitivity and reduce inflammation more effectively than either alone. The 12-week intervention in this study was effective, but longer-term studies with a greater sample size are needed to determine whether the benefits of exercise are sustained over time.

Limitations

The intervention period of 12 weeks may have been too brief to observe long-term effects. The study had a non-randomized allocation of participants. The study did not control for dietary intake or monitor participants’ nutrition, which may have influenced the outcomes. The study sample was relatively small, with only 80 participants, and the majority of the participants were female. This study compared aerobic and yoga separately but did not assess the effects of combined aerobic and yoga. ICAM-1 was selected as the sole marker of endothelial function due to its established relevance in vascular inflammation and its feasibility within our resource constraints; however, the inclusion of additional biomarkers such as VCAM-1 or functional assessments such as flow-mediated dilation would have provided a more comprehensive evaluation of endothelial health.

## Conclusions

This study highlights the remarkable benefits of aerobic and yoga training in improving endothelial function and insulin resistance in patients with T2DM. Aerobic exercise showed larger improvements in endothelial function, while yoga had larger improvements in insulin sensitivity. The findings demonstrate the utility of incorporating formal exercise into T2DM management as a non-pharmacologic intervention for decreasing cardiovascular risk and the progression of metabolic syndrome. There was no significant difference between the two exercise modalities, highlighting the fact that either type of exercise will lead to better cardiovascular health. Tailored exercises may be offered to physically fit patients as per their choice to reduce the complications of diabetes. These findings underscore the long-term potential of integrating structured aerobic and yoga-based exercise into routine diabetes care, not only to improve metabolic control but also to reduce the risk of vascular complications and enhance overall quality of life.

## Appendices

**Table 5:** Yoga resistance exercise protocol for individuals aged 18-35 years.

1. Prayer			1 minutes
2. Kriya: Kapalabhati		2 rounds of 30 strokes	2 minutes
3. Suryanamaskar		2 rounds	2 minutes
4. Yoga asanas			
Standing	Tadasana (the palm tree pose)	2 rounds	7 minutes
	Katichakrasana (the half-wheel pose)		
Sitting	Ushtrasana (the camel pose)		
	Sasakasana (the hare pose)		
	Vakarasana (the spinal twist pose)		
Prone	Dhanurasana (the bow pose)		
	Makarasana (the crocodile pose)		
Supine	Vipariti Karani (leg to the wall pose)		
	Halasana (the plough pose)		
	Saral Matsyasana (the fish pose)		
	Shavasana (the corpse pose)		
5. Pranayama		5 rounds	2 minutes
	Anuloma-Viloma (the alternate nostril breathing)		
6. Dhyana (breath awareness)		-	1 minutes
	Total duration		15 minutes

**Table 6:** Yoga resistance exercise protocol for individuals aged 35-50 years.

1. Prayer			1 minutes
2. Kriya: Kapalabhati		2 rounds of 30 strokes	2 minutes
3. Loosening practices		2 rounds	2 minutes
	Neck bending (forwards/backwards), shoulder rotation (clockwise/anticlockwise), trunk twisting (left and right)		
4. Yogasanas		2 minutes	7 minutes
Standing	Tadasana (the palm tree pose)		
	Hastottanasana (upstretched arms with side bending)		
	Padahastasana (the hands to feet pose)		
	Ardhacakrasana (the half-wheel pose)		
Sitting	Ardhaushtrasana (the half camel pose)		
	Sasakasana (the hare pose)		
	Uttanamandukasana (the stretched-up frog pose)		
	Vakrasana (the seated spinal twist pose)		
Prone	Sarala-Dhanurasan (the simple bow pose)		
Supine	Setubandhasana (the bridge pose)		
	Pavanamuktasana (the wind-releasing pose)		
	Shavasana (the corpse pose)		
5. Pranayama		5 rounds	2 minutes
	Anuloma-Viloma (the alternate nostril breathing)		
6. Dhyana (breath awareness)			1 minutes
	Total duration		15 minutes

**Table 7:** Yoga resistance exercise protocol for individuals aged 50-65 years.

1. Prayer			1 minutes
2. Kriya: Kapalabhati		2 rounds of 30 strokes	2 minutes
3. Loosening practice		2 rounds	2 minutes
	Neck movements		
	Forward and backward bending		
	Right and left bending		
	Right and left twisting		
	Shoulder movement		
	Rotation (clockwise and anticlockwise)		
	Trunk movement		
	Trunk twisting to left and right		
4. Yogasanas		2 rounds	7 minutes
Standing	Tadasana (the palm tree pose)		
	Ardha-katicakrasana (the lateral arch pose)		
Sitting	Dandasana (the staff pose)		
	Sukhasana (the easy pose)		
	Vakrasana (the seated spinal twist)		
Prone	Saral Bhujangasana/Bhujangasana (the cobra pose)		
	Ardha- Shalabhasana/Shalabhasana (single leg raise/both leg raise)		
	Makarasana (the crocodile pose)		
Supine	Markatasana (the monkey pose)		
	Ekpad Pavanamuktasana/Pavanamuktasana (the wind-releasing pose)		
	Shavasana (the corpse pose)		
5. Pranayama		3 rounds	2 minutes
	Anuloma-Viloma (the alternate nostril breathing)		
6. Pranayama			1 minutes
	Total duration		15 minutes
